# Case report: Cardiac arrest after radiofrequency ablation in a 76-year-old male

**DOI:** 10.1097/MD.0000000000037191

**Published:** 2024-02-23

**Authors:** Geya Jin, Shuyu Li, Yafeng Wang, Jianyi Pu

**Affiliations:** a North China University of Science and Technology, Tangshan, China; b Tangshan Workers’ Hospital, Tangshan, China; c Affiliated Hospital of North China University of Science and Technology, Tangshan, China.

**Keywords:** atrial fibrillation, case report, radiofrequency ablation, sinus arrest

## Abstract

**Rationale:**

Previous studies have found that the main treatment of sinus arrest is pacemaker treatment. It is rare to have 12 s of sinus arrest after radiofrequency ablation, and whether a permanent pacemaker is implanted immediately in this case is not described in the guidelines.

**Patient concerns:**

A 76-year-old male patient with persistent atrial fibrillation (AF) developed sinus arrest lasting 12 s in the early morning of the fourth day after using radiofrequency ablation for pulmonary vein isolation.

**Diagnosis:**

The patient was diagnosed with AF and sinus arrest.

**Interventions:**

The patient received cardiopulmonary resuscitation, intravenous injection of atropine 1 mg, and intravenous infusion of isoproterenol 1mg and immediately recovered consciousness thereafter. Approximately, 1.5 h later, the patient underwent surgery to install a temporary pacemaker in the right femoral vein.

**Outcomes:**

The patient had repeated episodes of sinus arrest after the implantation of a temporary pacemaker. After 3 weeks, the patient stabilized and was discharged. The patient was followed up for 1 year and did not experience any recurrence of sinus arrest or AF.

**Lessons:**

We consider the potential for postoperative myocardial edema, injury to the sinoatrial node during the procedure, propafenone poisoning, and autonomic dysfunction as contributors to the occurrence of sinus arrest after radiofrequency ablation. When sinus arrest occurs after radiofrequency ablation, we can choose the appropriate treatment according to the patient’s condition.

## 1. Introduction

Previous studies have found that the main treatment for sinus arrest is pacemaker treatment. It is rare to have 12 seconds of sinus arrest after radiofrequency ablation, and whether a permanent pacemaker is implanted immediately in this case is not described in the guidelines. We report a 76-year-old male patient who developed sinus arrest for 12 seconds in the early morning of the fourth day after radiofrequency ablation for pulmonary vein isolation to treat atrial fibrillation (AF).

## 2. Case report

A 76-year-old male came to our hospital for catheter ablation to treat persistent AF. The patient had a body mass index of 14.9 kg/m^2^ and reported a history of subclinical hypothyroidism. The patient denied any medical history of structural heart disease or hereditary arrhythmias. Self-reported chest tightness and palpitation were being treated by oral metoprolol taken within 30 minutes of onset for approximately 10 years. Oral cedilanid was added only approximately 1 month prior when metropolol alone stopped controlling the symptoms, but the drug combination was also ineffective. Subsequently, the patient was prescribed metoprolol 25 mg/d and diltiazem 45 mg/d to control arrhythmia; this regimen stabilized the heart rhythm and was stopped 2 days before the cardiac ablation.

At admission, the patient had a normal electrocardiogram, and myocardial enzymes, and N-terminal pro-brain natriuretic peptide was also normal. Preoperative transesophageal echocardiography showed that the left ventricular ejection fraction was normal and there was no thrombus in the left atrium or in the left atrial appendage. Atrial flutter and AF occurred intermittently several times while the patient awaited the procedure. The patient was sedated with fentanyl. Then, a 6F sheath with a 10-grade coronary sinus electrode catheter was inserted into the right femoral vein, and an atrial septal puncture needle and an atrial septal puncture sheath were placed along the femoral vein for atrial septal puncture. Pulmonary venography showed distortion of the pulmonary vein. The EnSite Precision Cardiac Mapping System was used to model the left atrium. A pressure catheter was used to isolate the bilateral pulmonary veins, and the left and right pulmonary veins were ablated at about 35 W and 43°C. A posterior large-head catheter was used to ablate the isthmus of the right atrial tricuspid valve. The patient experienced AF during the cardiac ablation. Lasso electrode detected fragmentation potential in the coronary sinus, but not in the left or right atrium. Lasso electrode mapping also indicated that the fragmentation potential in the superior vena cava was faster than in the coronary sinus; therefore, the superior vena cava was isolated, which slowed the central tachycardia and eventually restored sinus rhythm. Finally, bilateral pulmonary vein isolation and a 2-way block of the tricuspid isthmus were confirmed, and the procedure was ended.

**Figure 1.**
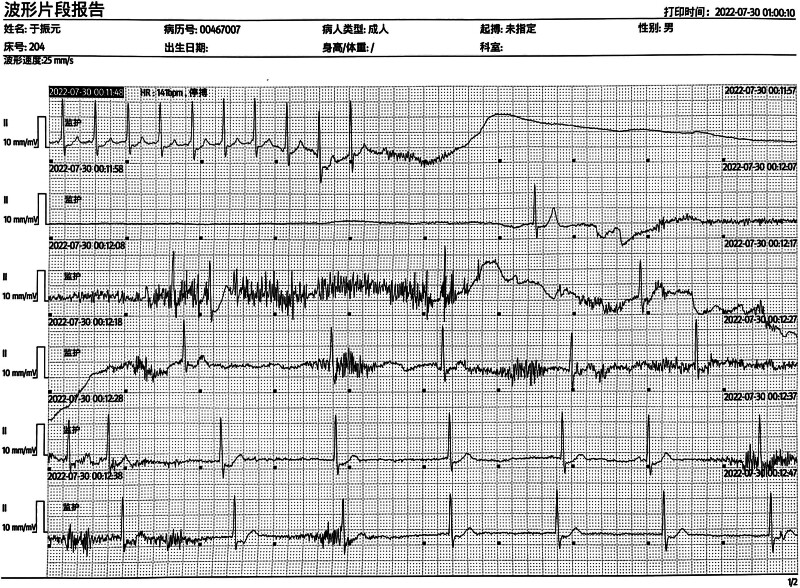
Sinus arrest and loss of consciousness occurred in the early morning of the fourth day (July 30, 2022) after ablation and lasted for up to 12 s.

After ablation, the patient had a CHADs2-VASc score of 2 and a HAS-BLED score of 1. Bloodwork showed elevated myocardial enzyme levels of myoglobin (91.65 ng/mL), cardiac troponin I (1.355 ng/mL), and creatine kinase isoenzyme (6.52 ng/mL). The patient was prescribed oral rivasaban 15 mg/d and propafenone 300 mg/d. On the second day after ablation, AF relapsed, and the patient’s heart rate dropped as low as 34 beats per minute (bpm). Propafenone was discontinued and amiodarone 0.6 g/d, XinBao pill 900 mg/d, and Shensong Yangxin capsule 3.6 g/d were administered. Sinus arrest and loss of consciousness occurred in the early morning of the fourth day after ablation and lasted for up to 12 seconds (Fig. 1). The patient received cardiopulmonary resuscitation, intravenous injection of atropine 1 mg, and intravenous infusion of isoproterenol 1 mg and immediately recovered consciousness thereafter. After regaining consciousness, the patient’s heart rate was 141 bpm, and he was experiencing atrial tachycardia and AF alternately. After several of these cycles, amiodarone was stopped, and a 5 mg dose of dexamethasone was injected intramuscularly. Approximately 1.5 hours later, the patient underwent surgery to install a temporary pacemaker in the right femoral vein. The pacemaker was set as follows: sensitivity threshold, 3.0 mV; pacing voltage, 5 mV; and pacing frequency, 70 bpm. The model and other details of the device were not recorded. Daily propafenone was administered at different doses in response to the patient’s status until stabilized. Drug doses and clinical events from the day of ablation through discharge are provided in Table 1.

**Table 1:** Drug doses and clinical events from the day of ablation (July 26, 2022) through discharge.

	Propafenone	β-Blocker (metoprolol tartrate)	Amiodarone	Diltiazem	Number of times of atrial fibrillation	Number of long R-R intervals (>3.0 s)	The longest time of long R-R interval
Day 1 July 27, 2022	300 mg	0	0	0	0		
Day 2 July 28, 2022	0	12.5 mg	0.75 g	0	5	0	/
Day 3 July 29, 2022	0	12.5 mg	0.6 g	0	0	2	12.0 s (in the early morning of July 30)
Day 4 July 30, 2022	0	12.5 mg	0	60 mg	5	0	/
Day 5 July 31, 2022	100 mg	0	0	50 mg	4	1	7.6 s
Day 6 August 1, 2022	150 mg	0	0	0	0	0	/
Day 7 August 2, 2022	150 mg	0	0	0	0	0	/
Day 8 August 3, 2022	150 mg	0	0	0	0	0	/
Day 9 August 4, 2022	225 mg	0	0	0	1	1	6.0 s
Day 10 August 5, 2022	225 mg	0	0	0	0	0	/
Day 11 August 6, 2022	225 mg	0	0	0	0	0	/
Day 12 August 7, 2022	225 mg	0	0	0	0	0	/
Day 13 August 8, 2022	225 mg	0	0	0	0	0	/
Day 14 August 9, 2022	300 mg	0	0	0	1	0	/
Day 15 August 10, 2022	300 mg	0	0	0	1	0	/
Day 16 August 11, 2022	300 mg	0	0	0	0	0	/
Day 17 August 12, 2022	300 mg	0	0	0	1	0	/
Day 18 August 13, 2022	400 mg	0	0	0	5	0	/
Day 19 August 14, 2022	600 mg	0	0	0	4	0	/
Day 20 August 15, 2022	600 mg	0	0	0	4	0	/
Day 21 August 16, 2022	600 mg	0	0	0	0	0	/
Day 22 August 17, 2022	600 mg	0	0	0	0	0	/

## 3. Discussion

Tachy-brady syndrome (TBS) is one of the many classifications of sick sinus syndrome. TBS is often characterized by long-term sinus arrest after a sustained period of rapid atrial rhythm. Rapid atrial arrhythmia after sinus arrest longer than 3 seconds is usually AF. In most patients with TBS, the duration of sinus arrest is relatively short; however, sinus arrest lasting 12 seconds has been documented. The notable finding in this case of sinus arrest is that there were no clinical manifestations of TBS, atrioventricular block, or sinoatrial node disease before radiofrequency ablation; rather, TBS and sinus arrest occurred shortly after radiofrequency ablation. We note 4 factors that probably contributed to this post-procedure occurrence of fast–slow syndrome and sinus arrest.

### 3.1. Postoperative myocardial edema

First, we posit that postoperative myocardial edema may have contributed to the development of TBS and sinus arrest. Previous studies have shown that myocardial edema can occur within 1 day after radiofrequency ablation.^[1–7]^ The degree of edema depends on the intensity and quantity of radiofrequency energy during pulmonary vein ablation.^[8]^ The fluid is usually absorbed in about 1 to 3 months,^[3,9,10]^ and the speed of resorption is closely related to early recurrence of AF,^[11]^ which may be due to changes in conductance and transmembrane ion currents soon after ablation or to cardiomyocyte edema.^[12–19]^

The effect of aquaporins on cardiac electrophysiology is not clear. Aquaporins on the cell membrane are permeable to water and, therefore, may indirectly affect electrophysiology by potentiating the swelling or contraction of cardiomyocytes, thereby altering the ion concentration in the intracellular and extracellular fluids.^[15]^ Alternatively, aquaporins may interact with other membrane proteins or form macromolecular complexes to affect cardiac electrophysiology.^[20]^

Usually, there are no serious complications related to postoperative cardiac edema. However, Steel et al^[21]^ reported a case of severe left atrial edema, which persisted for 2 months after pulmonary vein ablation and led to atrial mechanical stunning and severe heart failure. Left atrial wall edema may lead to thrombosis; therefore, after pulmonary vein ablation, anticoagulation therapy should be given for at least 1 month, even if there is no recurrence of AF. In this case, the patient experienced postoperative increase in myocardial enzymes, indicating the presence of myocardial injury as well as the onset of myocardial edema. Early treatment with corticosteroids may accelerate resorption of fluids.

### 3.2. Sinus node dysfunction

For this patient, ablation was accomplished by isolation of the bilateral pulmonary vein and vena cava. The ectopic electric activity of the superior vena cava is the most common origin of non-pulmonary venous AF, but radiofrequency ablation of the SVC is associated with a risk of sinus damage.^[22]^ During SVC isolation, sinoatrial node injury is defined as sinoatrial node pausing for >3 seconds, marked sinus bradycardia, or persistent slow junctional rhythm with atropine and/or dopamine excluding vagus nerve reflex.

In this case, the dynamic electrocardiogram performed before radiofrequency ablation excluded pathological sinus syndrome, but sinus arrest for up to 12 seconds occurred post-operatively. Intravenous atropine was administered after the sinus arrest, and the patient’s heart rate reached a maximum of 141 bpm, considering the vagal effect. Injury to the sinoatrial node has been reported after SVC isolation procedures. Chen et al^[22]^ summarized the most important reasons for the high incidence of this injury: rapid reflux of the contrast medium during radiography can cause the sinoatrial node injury; mis-identification of the location of the SVC and the sinoatrial node and the displacement of the ablation catheter, which can be mitigated by precisely defining the junction of the right atrium and SVC by intracardiac echocardiography and performing the SVC isolation during stable sinus rhythm; some surgery centers do not possess intracardiac color Doppler ultrasound to precisely identify the right atrium SVC; the location of the sinoatrial node cannot be identified during sinus rhythm, nor can the function of the sinoatrial node be evaluated, so postoperative complications caused by sinoatrial node injury cannot be excluded; and displacement of the ablation catheter.

### 3.3. Drug effects

It has been shown that the administration of anti-arrhythmia drugs soon after radiofrequency ablation can significantly reduce the early and long-term recurrence rate of AF. Amiodarone and propadenone are 2 common anti-arrhythmics, with the former being more commonly prescribed. In this case, the patient was prescribed propadenone to control arrhythmia after surgery but experienced postoperative atrial arrhythmia and sinus arrest. We cannot rule out that the frequent changes in the patient’s anti-arrhythmia medications may have contributed to these outcomes, and the patients medication history should be considered when selecting postoperative anti-arrhythmia drugs. However, further research is needed to more precisely define the strategies for selecting anti-arrhythmia drugs after radiofrequency ablation.

### 3.4. Pacemaker implantation

The use of a permanent pacemaker to prevent potential sinus arrest after AF is controversial. This patient developed severe sinus arrest after surgery, which resolved upon placement of a temporary pacemaker and continued to prevent sinus arrest during the 1-year follow-up period. This sinus arrest that occurred during the blanking period may be related to the following: edema of the tissue surrounding the sinus atrial node; degenerative changes (sinus node remodeling) caused by prolonged AF; postoperative administration of drugs to control arrhythmia affecting sinus node self-regulation; and/or increased vagal tone due to severe sinus arrest at night. We thought the sinus dysfunction that occurred during the blanking period was probably reversible, and we installed a temporary pacemaker, which was less resource-intensive compared with actively inserting a permanent device. The temporary pacemaker effectively reduced the patient’s pain and improved quality of life, with effects lasting for at least 1 year.

In the event of sinus arrest after radiofrequency ablation, whether to implant a permanent pacemaker immediately is not described in the guidelines. In the past, doctors would implant permanent pacemakers immediately according to experience to ensure the safety of patients. We recorded that when sinus arrest occurred in the elderly after radiofrequency ablation with a variety of antiarrhythmic drugs, we did not immediately implant a permanent pacemaker, but implanted a temporary pacemaker to continue to observe the patient’s condition. After a year of follow-up, the patient did not have sinus arrest again, and the prognosis was good, which can provide experience for similar clinical situations in the future.

This article has the following limitations: First, this patient does not have perfect coronary angiography to determine whether the right coronary artery is narrow, and it is impossible to determine whether sinus arrest is caused by sinoatrial node disease or coronary artery occlusion. In addition, it is only a case report with a small number of cases and does not form a case-control study. In the future, we can form a medical record control study on whether to implant a permanent pacemaker to understand the long-term prognosis of patients. If patients do not need a permanent pacemaker, it can reduce the cost and improve the quality of life.

## 4. Conclusion

We consider the potential for postoperative myocardial edema, injury to the sinoatrial node during the procedure, propafenone poisoning, and autonomic dysfunction as contributors to the occurrence of sinus arrest after radiofrequency ablation. As illustrated in this case, the patient’s previous medication should be considered in order to more accurately identify antiarrhythmic drugs after radiofrequency ablation and should be on guard against the occurrence of sinus arrest. When sinus arrest occurs after radiofrequency ablation, we can choose the appropriate treatment according to the patient’s condition.

## Author contributions

Data curation: Geya Jin, Yafeng Wang.

Investigation: Geya Jin, Yafeng Wang.

Writing—original draft: Geya Jin, Yafeng Wang.

Supervision: Shuyu Li, Jianyi Pu.

Writing—review & editing: Shuyu Li, Jianyi Pu.
